# Endoscopic ultrasound-guided hepaticogastrostomy for patients with frequent respiratory fluctuations using a novel hybrid guidewire to prevent guidewire shearing

**DOI:** 10.1055/a-2378-6319

**Published:** 2024-08-16

**Authors:** Takeshi Ogura, Yuki Uba, Nobuhiro Hattori, Jun Matsuno, Hiroki Nishikawa

**Affiliations:** 1 13010 2nd Department of Internal Medicine, Osaka Medical and Pharmaceutical University, Osaka, Japan; 2 38588 Endoscopy Center, Osaka Medical and Pharmaceutical University Hospital, Osaka, Japan

Endoscopic ultrasound-guided hepaticogastrostomy (EUS-HGS) is widely attempted in patients after failed endoscopic retrograde cholangiopancreatography (ERCP) 1 2 . EUS-HGS requires several procedural steps, including bile duct puncture, guidewire insertion, tract dilation, and stent deployment. Among these procedural steps, guidewire insertion may be most challenging, especially in nonexpert hands 3 . A liver impaction technique may be useful for improving the technical success rate of guidewire insertion 4 5 ; however, this technique often cannot be performed, especially in patients with nondilated bile ducts or frequent respiratory fluctuations, because guidewire visualization on EUS images can be difficult.

To prevent guidewire shearing, improvements in the guidewire are needed. Guidewires can be divided into two types: polytetrafluoroethylene (PTFE) press coating or jacket type. Generally, although a PTFE press coating can prevent guidewire shearing, the resultant resistance due to friction can be high compared with jacket-type guidewires ( Fig. 1 ). In the presence of bile juice or contrast medium, it may therefore not be possible to smoothly perform guidewire manipulation or device exchange. To overcome this issue, a novel hybrid guidewire (CAPELLA; Japan Lifeline Co., Ltd., Tokyo, Japan) has recently become available. This guidewire is made by PTFE press coating the guidewire from the tip to a distance of 195 mm; the remaining part is the jacket type with grooving ( Fig. 2 ). This guidewire serves two purposes: preventing guidewire shearing and decreasing friction resistance. When there are frequent respiratory fluctuations, guidewire shearing can be frequent complication, meaning several techniques, such as the liver impaction technique, may not be feasible. The characteristics of the guidewire are therefore important.

**Fig. 1:**
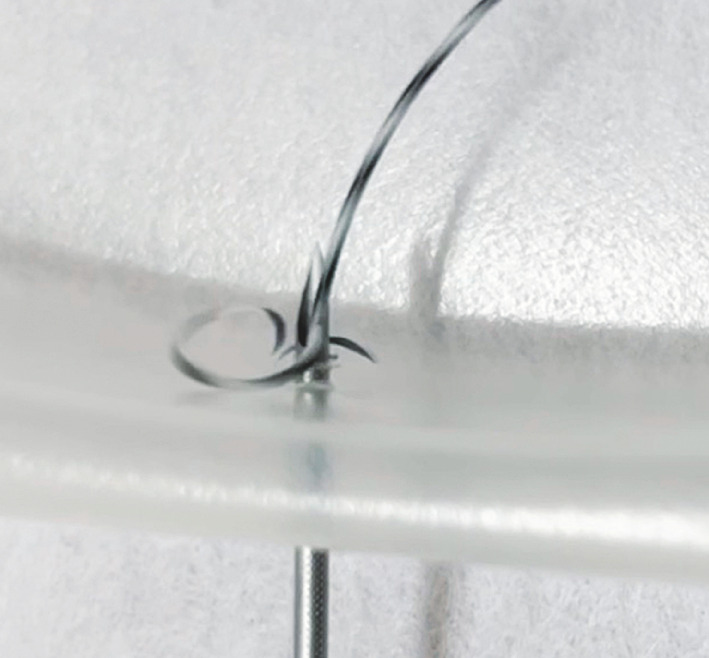
Photograph showing a jacket-type guidewire that has been sheared by the needle as can happen during guidewire manipulation.

**Fig. 2:**
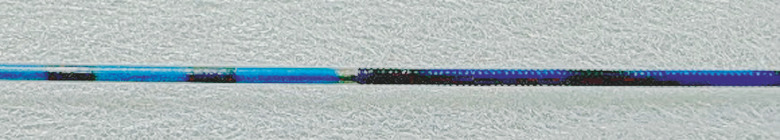
Photograph of the novel hybrid guidewire (CAPELLA; Japan Lifeline Co., Ltd., Tokyo, Japan), which is made with a polytetrafluoroethylene press coating from the tip to a distance of 195 mm, with the remaining part being of the jacket type with grooving.

Herein, we describe technical tips for performing EUS-HGS when there are frequent respiratory fluctuations using this novel hybrid guidewire. First, bile duct puncture was successfully performed using a 19G needle and the contrast medium injection was carefully performed with attuning respiratory fluctuations ( Fig. 3 **a** ). The novel hybrid guidewire was then successfully inserted and manipulated to deploy it deeply without guidewire shearing ( Fig. 3 **b** ). After tract dilation using a balloon catheter, a metal stent was successfully deployed without any adverse events ( Fig. 3 **c** ; Video 1 ).

**Fig. 3:**
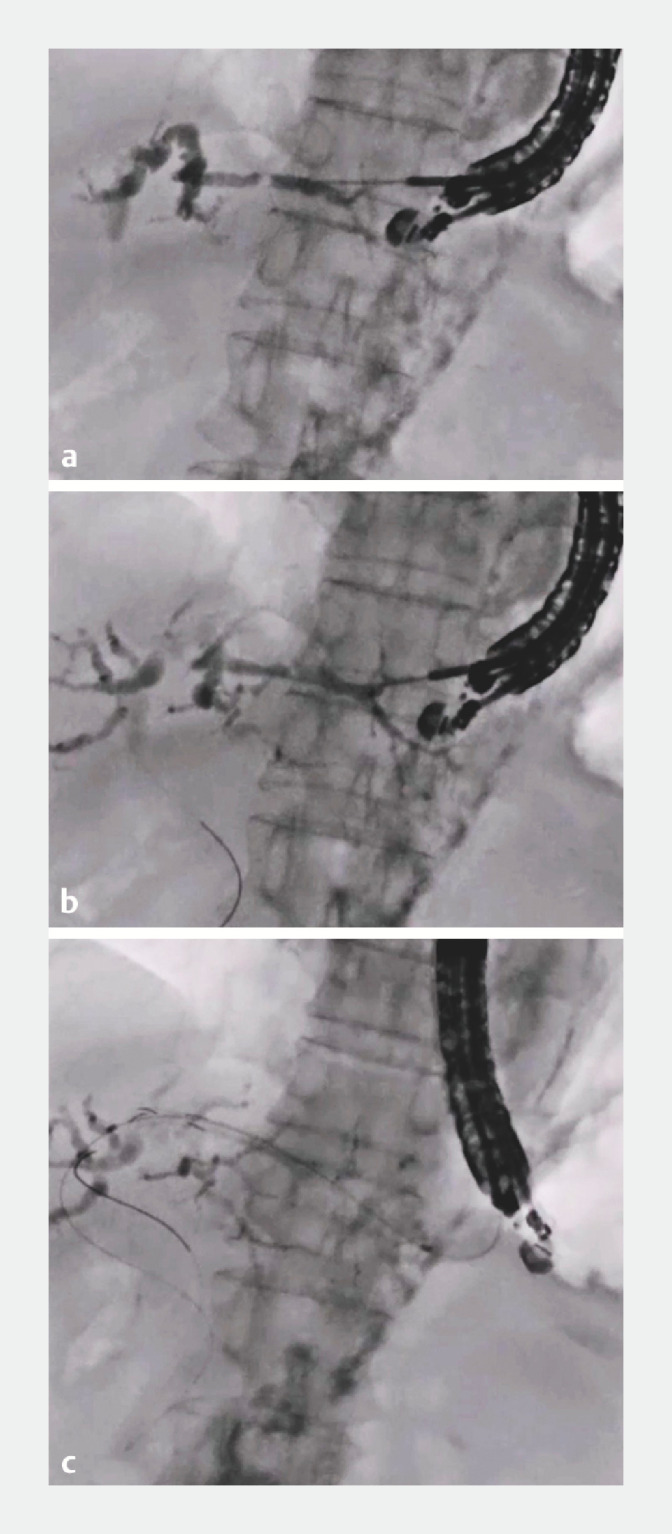
Fluoroscopic images showing: **a** bile duct puncture and contrast medium injection being performed; **b** the novel hybrid guidewire being deployed; **c** deployment of the metal stent.

Endoscopic ultrasound-guided hepaticogastrostomy is performed in a situation where there are frequent respiratory fluctuations using the novel hybrid guidewire.Video 1

In conclusion, EUS-HGS using this novel hybrid guidewire may be helpful for preventing guidewire shearing in cases where application of the liver impaction technique is difficult.

Endoscopy_UCTN_Code_TTT_1AS_2AH

## Notes

Endoscopy E-Videos https://eref.thieme.de/e-videos
*E-Videos* is an open access online section of the journal *Endoscopy* , reporting on interesting cases and new techniques in gastroenterological endoscopy. All papers include a high-quality video and are published with a Creative Commons CC-BY license. Endoscopy E-Videos qualify for HINARI discounts and waivers and eligibility is automatically checked during the submission process. We grant 100% waivers to articles whose corresponding authors are based in Group A countries and 50% waivers to those who are based in Group B countries as classified by Research4Life (see: https://www.research4life.org/access/eligibility/ ). This section has its own submission website at https://mc.manuscriptcentral.com/e-videos .
